# TIGIT predominantly regulates the immune response via regulatory T cells

**DOI:** 10.1172/JCI183278

**Published:** 2024-07-01

**Authors:** Sema Kurtulus, Kaori Sakuishi, Shin-Foong Ngiow, Nicole Joller, Dewar J. Tan, Michele W.L. Teng, Mark J. Smyth, Vijay K. Kuchroo, Ana C. Anderson

Original citation: *J Clin Invest*. 2015;125(11):4053–4062. https://doi.org/10.1172/JCI81187

Citation for this retraction: *J Clin Invest*. 2024;134(13):e183278. https://doi.org/10.1172/JCI183278

QIMR Berghofer Medical Research Institute recently notified the *JCI* of concerns regarding Figure 4B and Figure 7B and indicated that an independent panel investigation concluded that these figures are likely based on data fabricated or falsified by Mark J. Smyth. In addition, the investigative panel concluded that Figure 7A is based on unreliable data produced by Mark J. Smyth. The QIMR Berghofer Medical Research Institute investigation found that one author, Mark J. Smyth, was the sole individual responsible for provision of data in Figure 4B, Figure 7A, and Figure 7B. Owing to these data integrity concerns, the *JCI* is retracting this article. No issues were raised with regard to any of the other data in the article.

Ana C. Anderson and Vijay K. Kuchroo have agreed with the Journal’s decision to retract the paper. The remaining authors abstained from commenting or could not be reached.

## Version 1 07/01/2024

Electronic publication

